# What is improvement science, and what makes it different? An outline of the field and its frontiers

**DOI:** 10.3389/frhs.2024.1454658

**Published:** 2025-02-20

**Authors:** Julie E. Reed, Grazia Antonacci, Natalie Armstrong, G. Ross Baker, Sonya Crowe, Karin Pukk Harenstam, Dougal Hargreaves, Yogini H. Jani, Lloyd Provost, Martin Rejler, Carl Savage, Johan Thor, Sharon Williams, Thomas Woodcock

**Affiliations:** ^1^ School of Health and Welfare, Halmstad University, Halmstad, Sweden; ^2^ Julie Reed Consultancy Ltd., London, United Kingdom; ^3^ School of Public Health, Imperial College London, London, United Kingdom; ^4^ Department of Population Health Sciences, University of Leicester, Leicester, United Kingdom; ^5^ Institute of Health Policy, Management and Evaluation, University of Toronto, Toronto, ON, Canada; ^6^ Clinical Operational Research Unit, University College London, London, United Kingdom; ^7^ Medical Management Centre, Karolinska Institutet (KI), Solna, Sweden; ^8^ Imperial College London, London, United Kingdom; ^9^ Centre for Medicines Optimisation Research & Education, University College London Hospitals NHS Foundation Trust, London, United Kingdom; ^10^ School of Pharmacy, University College London, London, United Kingdom; ^11^ Associates in Process Improvement, Austin, TX, United States; ^12^ School of Health and Welfare, Jönköping University, Jönköping, Sweden; ^13^ The Jönköping Academy for Improvement of Health and Welfare, Jönköping, Sweden; ^14^ School of Health and Social Care, Swansea University, Swansea, United Kingdom

**Keywords:** improvement science, quality improvement, healthcare, complex system, implementation sceince, patient safety, knowledge mobilisation

## Abstract

Improvement science has emerged as an interdisciplinary field of enquiry to provide methodological and scientific rigour to the practice and study of improvements in healthcare, and with contributions from a wide range of stakeholders and perspectives. However, compared to more well-established health-related sciences, the science of improvement remains in relative infancy. Whilst the improvement community has grown considerably, there is no existing articulation of the scope of what matters to the health and social care improvement community, and how this aligns to the enquiries of the field of improvement science. This paper aims to outline key areas of interest to the improvement community, and to propose distinguishing features of improvement science that help differentiate it from other areas of enquiry. Two over-arching research questions are identified, along with ten associated areas of enquiry which are grouped into three clusters: (1) improvement in practice, (2) aligning improvement efforts and (3) advancing the contribution of the improvement community. Four features that collectively define and distinguish the field of improvement science are proposed. The outline of the improvement landscape provides a common language for the diverse improvement community, supporting people to transcend disciplinary interests and constraints, and to consider how, collectively, we can improve health and care. Others are invited to refine and advance mapping of the improvement landscape by identifying gaps and increasing contributions from diverse perspectives.

## Introduction

Understanding how to improve healthcare in different settings and diverse contexts is a major global challenge to ensure the fundamental rights of every human being to “the enjoyment of the highest attainable standard of health” (1).

Improvement science has emerged in response to these challenges, seeking to provide methodological and scientific rigour to the practice and study of improvements in healthcare, and engaging contributions from a wide range of stakeholders and perspectives. However, compared to more well-established health-related sciences, the science of improvement remains in relative infancy and was described by Marshall et al. as being in the “pre-paradigm” phase due to the lack of agreed definition of the field (2). Khun defines a scientific paradigm as a community with a shared focus on “what matters”:

“There is room for considerable disagreements within such a field, often about very basic issues. What is shared, however, is a sense of what is at issue, why it matters, and what must be done to resolve it” (2).

Whilst the improvement community has grown considerably since Marshall's 2013 publication, to the best of our knowledge there is no existing articulation of the scope of what matters to the health and social care improvement community, and how this aligns to the enquiries of the field of improvement science.

This paper aims to outline key areas of interest to the improvement community, and to propose distinguishing features of improvement science that help differentiate it from other areas of enquiry. Our intent is that by articulating *what matters*, this paper can contribute to advancing the science of improvement beyond its “pre-paradigm” phase.

Defining the scope and interests of the improvement community is complicated by the various ways in which the term improvement science has been adopted by different groups, meaning that even the term “improvement science” is contested (3). For example, the term improvement science can be used:

– To represent the classic schools of thought that emerged in non-healthcare industries that provide mental models for thinking about improvement in systems (e.g., Demings system of profound knowledge) (4, 5).
– As a synonym for the term “quality improvement”, with the implication that any given suite of tools and methods utilised for quality improvement (e.g., six sigma, lean, model for improvement) provide a structured scientific approach to make improvements in practice (6).
– To describe the study and evaluation of quality improvement approaches in practice (i.e., to build evidence about what quality improvement approaches work, when, for whom and for what purposes) (7–9).
– To consider the philosophical assumptions of sciences that aim to achieve improvements and their comparison and contrast to more traditional sciences that aim to build knowledge (10–12).

Rather than advocating for one or another of these perspectives on what improvement science is, we believe that all perspectives have value to add. Being explicit about these contributions will help to widen people's awareness of the breath of what the field of improvement science can include, and how these different interpretations support, compliment and at times conflict with each other. It is beyond the scope of this paper to define and lay out all of the factors that need to be considered in this respect—but we invite others to contribute to discussion and reflections on these issues as part of this special issue.

## Background

As a group of authors we had the privilege of being exposed to diverse contributions to the study and practice of improvement through our engagement in an international improvement science fellowship community (see Supplementary Material for further details). Through these interactions we became aware that, as an emerging field, improvement science is still defining its scope and its differentiation from other fields of enquiry and practice. There is no doubt that improvement science has been influenced by and draws heavily upon more established fields including patient safety, quality improvement, implementation science, innovation and intervention research, health service research, health economics, complex systems thinking, sociology, psychology, operational research, engineering, design, organisational and management sciences to name but a few.

Given the extensive contributions from and overlap with interests of other fields, common questions we encountered were “what is actually new in the field of improvement science?”, and “how is it different from what has gone before?”.

In our growing appreciation of the diversity of perspectives in the improvement community, we also became aware of the risk that members of the improvement community work in relative isolation, focused on specific areas of enquiry, and opportunities for collaboration and enhanced overall understanding are missed. The diversity of the improvement community reflects the complexity and intricacy of the challenges faced. As well as attracting researchers from various disciplines, patients, practitioners, clinical leaders, educators, management consultants, industry partners, and policy makers, among others, are also key contributors to the improvement community. These different backgrounds influence where people's interests lie, and provide different ways of framing challenges and explaining phenomena along with their associated tools, methodologies, and perspectives—all of which can make it challenging to communicate and collaborate with other members of the improvement community.

As such, we saw value in developing an outline of the improvement landscape to: articulate the breadth of areas that matter to members of the improvement community; illustrate key questions that are at the frontiers of the field of improvement science; and propose a series of features that distinguish improvement science from other fields of enquiry.

First and foremost, we believe that initiating an outline of the field of improvement science and proposing distinguishing features of the field will help to create a common language to foster collaboration and help the community to grow and achieve its goals.

Second, by highlighting questions at the frontiers we leave scope for contributions from different perspectives to address them, enabling people to transcend disciplinary interests and constraints, and to consider how, collectively, we can improve health services.

Third, there is a need to clearly articulate the improvement landscape to be able to communicate and gain traction with those outside of the current improvement community, including other academic disciplines, healthcare leaders, managers and practitioners, funders, governments and the general public.

Finally, we propose that the provision of a structured outline of areas of enquiry can inform decisions concerning the design, conduct and evaluation of improvement initiatives and research enquiries, and inform the development of capacity and capability of improvement community members.

## Process of development

The outline of the improvement landscape was developed by drawing on the breadth of our professional backgrounds as authors, and in consultation with the international improvement community. An initial outline of the improvement landscape was developed by the authorship group. It was then further expanded and refined in an iterative process with feedback obtained from the wider improvement community in an opportunistic manner through presentations and workshops delivered by the authorship group as part of existing national and international conferences (the Improvement Science and Research Symposium, UK, International Forum on Quality and Safety in Healthcare, UK; Health Service Research conference, UK; The International Society for Quality in Health Care (ISQua) Malaysia; IHI National Forums Scientific Symposium, USA) and in partnership with the international Improvement Science Development Group supported by the Health Foundation, UK. These community participants had a variety of roles within the health system (e.g., academics, healthcare professionals, quality improvement experts, managers, policy makers, patients, etc.). In total, over 380 stakeholders were involved (with groups ranging from 20 to over 100 participants). Feedback was collected via a mixture of group discussions and electronic feedback (using Mentimeter software). Analysis focused on developing a comprehensive overview of the breadth of concepts raised, and identifying themes to logically group and capture the range of topics discussed. Further details about the process are provided in the Supplementary Material.

In discussions with stakeholders, we focused on topics relating to *how* to achieve improvement in care, rather than *what* needs to be improved. We deliberately did not include the specific areas of health, health services, population health, or societal concerns that need improving and are covered extensively by clinical groups, public health and international bodies (13). The outline of the improvement landscape therefore contains generic issues we believe are pertinent to the improvement of any aspect of health and social care.

We made a concerted effort to value different perspectives and vocabularies and invested time to understand the diverse theories and disciplines, rather than negotiating away nuance in favour of quick compromise. We paid particular attention to the use of language that was accessible and enabled diverse disciplines to engage.

## An outline of the improvement landscape

In reviewing feedback about *what matters* to members of the health service improvement community, we identified two over-arching questions, and ten associated areas of enquiry (Table 1).

**Table 1:** An outline of the improvement landscape.

Cluster	What matters to the improvement community: areas of enquiry	Illustrative questions at the frontiers of improvement science
Improvement in Practice	Achieving improvement • Initial impact • Sustainability • Spread	How do different people define what an ‘improvement’ is, and how are competing priorities and tensions taken into account when setting direction for improvement? How to implement changes and achieve initial impact on health and wellbeing of improvement efforts within specific contexts? How to sustain improvement for long term success? How to achieve scale and spread of successful improvement beyond initial contexts?
	Approaches to achieving improvement • Contribution of an approach (strategies, methods and tools) to improvement • Doing an approach well • Supporting systemic use of an approach • Building supportive organisational culture	What problems or questions can different approaches help resolve, at what cost, and how do we know? What aspects of an approach are important, and how can they be applied with fidelity in practice? How can meaningful coproduction be practised to achieve person centred care? How can improvement approaches be designed and utilised effectively at different system levels? How can cultural, structural and process factors be optimised at different system levels to support a given approach?
	Integrating improvement in context • Improvement as core business • Competing agendas and priorities • Awareness of system complexity • Adapting and responding to real world pressures and in times of crisis	How might teams, organisations and systems integrate improvement with other priorities in daily work? Can improvement become a continuous process supplemented with discrete projects? How can organising and learning systems be established to enable continuous improvement? How can useful research be produced that takes into account the complex, fast changing and political nature of healthcare within and across system levels? How can research and practice anticipate and adapt to the emergent nature of improvement work and healthcare systems, including at times of crisis?
	Measurement and evaluation to inform improvement • Understanding the system • Informing the process of improvement • Evaluation • Improving measurement and evaluation	How can measurement and evaluation be used to guide and learn from improvement efforts at team, organization and system levels? How do we advance measurement to make it easier to enact in practice? How do we build organisational and system infrastructure to support measurement? How do we conduct evaluation of complex interventions in complex social systems to produce knowledge that is useful for future improvement and decision making? How can unintended consequences of measurement be mitigated?
Aligning improvement efforts	The interdependence of system levels • Micro level • Meso level • Macro level	How do we understand and coordinate improvement initiatives within and across each system level? How do we navigate the influence of the social, political and cultural context on improvement efforts within and across different system levels? How can we optimise structures, processes and roles to better support system coordination?
	The interactional work of improvement • Relationships, trust, respect • Influencing skills • Facilitation • Motivation • Vested interests, power dynamics • Navigating uncertainty and contingencies	How can we create meaningful collaborations and partnerships between diverse stakeholders, including patients, communities and seldom heard groups, to understand what matters to them, and to align improvement with intrinsic and extrinsic motivators with cultural sensitivity? How can we better understand and articulate the hidden interactional work of improvement? How can we create a culture where interactional work is valued and where building the appropriate skills and competencies is promoted?
	Enhancing the value of improvement practice and research • Benefit vs cost/harm • Resource allocation • System efficiency	How can we choose wisely to invest (or disinvest) resources, time and expertise to maximise the value of improvement efforts, and of activities to improve the way we improve? How can a balance be struck between the pace of practice and the rigour of research, and if so, can we agree what is good enough? How can we optimise investment in improvement research and practice across a health system and all relevant stakeholders?
Advancing the Contribution of the Improvement Community	Seeking synergies between knowledge, research, and practice • Building on existing knowledge • Improving learning in practice • Synergies between improvement practice and research	How can we ensure that research addresses issues that are of importance to patients and practice and produces findings that are useful and actionable? How can we generate useful and relevant knowledge from practice including from planned and natural experiments? How can practice and research stakeholders collaborate to ensure that knowledge translates into practice to facilitate improvement? How can we organise and coordinate research and practice across and within different system levels to promote learning and improvement?
	Ways of knowing and doing • Axiology – what values motivate and shape improvement research and practice? • Ontology - what is there to know? • Epistemology - how can you know? • Methodology - how do you generate knowledge?	How can researchers and practitioners better understand their own and others’ philosophical perspectives? How can researchers and practitioners be supported in selecting appropriate philosophical and methodological approaches for research and practice of improvement? How can communication between stakeholders be facilitated so that different approaches and knowledge contributions are valued based on their merits and limitations? How can we create an evidence base for the value and limitations of different ways of knowing and doing, to inform appropriate selection for practice, research and funding decisions?
	Building the community • Education and professional development • Developing future leaders • Collaborative working • Making and communicating a distinctive and externally appreciated contribution to healthcare	How can stakeholders be attracted and supported to develop capability and propensity to collaboratively improve healthcare? How can a new generation of leaders be developed with the skills, knowledge, values and vision to lead research and practice across the boundaries of current systems? How can effective teaching and learning approaches at all system levels be developed and organised for improvement practice and research? How can improvement scientists increase awareness, interest and support from academia, practice, policy and the public?

The two over-riding questions that matter to the improvement community are:

• “How can we achieve improvements in complex systems?”
• “How can we improve the way we improve?”

The ten areas of enquiry vary in character from the practical to the more philosophical. Whilst each area of enquiry is presented as somewhat discrete, there are important interconnections, overlaps, and tensions. This led us to group the areas of enquiry into three clusters: (1) Improvement in Practice (2) Aligning Improvement Efforts (3) Advancing the Contribution of the Improvement Community.

Within each area we identified key questions that illustrate issues of major interest to the field of improvement science (Table 1). Many of these questions have been partially addressed by contributions in the academic literature or by examples in practice. However, none of them have been fully resolved and barriers remain to effective application and spread of improvement knowledge. For example, there is evidence of both successful and unsuccessful attempts at using various quality improvement approaches to support change, but it is not yet clear which approaches are best suited for different problem types, and in different contexts (14). Evidence also demonstrates that quality improvement approaches are not used with high fidelity, but it is not yet clear how to reliably improve the fidelity of their use in diverse settings (15). Therefore, all questions represent areas at the frontiers of current knowledge, with active and ongoing enquiry in research and practice.

## Cluster 1—improvement in practice

### Achieving improvements

The first area reflects the focal point that all other areas are working towards, the desired outcome of improvement efforts. People work to improve different facets of quality of care including safety, efficiency, effectiveness, timeliness, patient-centeredness, staff-wellbeing, and equality (16–18). Whole fields of study have developed to explore improving aspects of quality in more detail, including patient safety focusing on the reduction of harm, implementation science focusing the delivery of evidence-based care, and coproduction focusing on patient and person centred care. Other areas have received notably less attention such as equality and equity of health services, although this is gradually changing. Central to this area of enquiry is the question of what is being improved, who sets the agenda, and the trade-offs and balance between different facets of quality e.g., efficiency and effectiveness. Despite advances in understanding of how to achieve improvements, challenges still remain in knowing how to reliably achieve an impact on care quality and how improvements can be sustained. In addition there are recognised challenges to the spread and reproduction of successful initiatives beyond their initial setting, including understanding how to adapt interventions and initiatives to work in different contexts (19, 20).

### Approaches to achieving improvement

In seeking to achieve these quality gains people have explored the use of a wide range of quality improvement approaches (e.g., quality improvement methods, national quality registries, audit and feedback, policy changes). Research is exploring the impact and mechanisms of action of different approaches. This includes understanding their benefits and limitations, knowing what approaches to use in different settings and for different improvement goals, and how approaches can be optimised for greatest impact in healthcare settings. It has been demonstrated that approaches are adopted with varying degrees of fidelity (21) and that effective use is influenced by organisational context including infrastructure, leadership and learning culture (22). As such there is a need to understand how best to support the approaches for optimal use, including understanding how to improve fidelity of use, understanding what aspects of an approach are most important to adhere to for successful use, and how approaches can be adapted to work in a variety of settings and constraints. This understanding is necessary to support the successful application, evaluation, attribution, spread and reproducibility of improvement approaches (23).

### Integrating improvement in context

Improvement takes place alongside “business as usual” and is influenced by institutional factors such as staffing, busy-ness, and the need to respond to crises such as the Covid-19 pandemic. We need to understand how improvement can become an integral, core activity, and how it fits with the daily practice, operational management and strategic planning of health services. Consideration needs to be given to how improvement initiatives and improvement approaches are experienced by healthcare staff including understanding their prior experiences and receptivity to improvement work, the extent to which initiatives align with or compete with other responsibilities, and how to manage time constraints for improvement work alongside the delivery of care. Improvement approaches and research studies needs to be optimised to work in complex and dynamic health care systems (24), recognising the fast pace of change and emergent issues that require consistent adaptation and revised responses. Understanding how to build individual, organisational and system resilience to adapt and align improvement activities to a constantly changing workplace is essential to embed and sustain improvements (20).

### Measurement and evaluation to inform improvement

Critical to all improvement efforts is the need to know whether the changes made have resulted in the desired improvement, and what other intended or unintended consequences the changes have resulted in. It is also desirable to know how an initiative has achieved improvements, to support effective implementation, and learn how to reproduce success in other settings. For instance, theory and methods developed by Walter Shewhart have been used effectively to inform and support improvement efforts (25, 26). This approach differs from statistical approaches more traditionally used in research, such as frequentist hypothesis testing, in accepting and quantifying the variation inherent to the messy reality of health systems, rather than attempting to isolate one cause of variation, enabling teams to identify important signals in data in close to real time, to inform and support improvement efforts. Existing methods of measurement and evaluation are often poorly used, and frequently do not answer the salient questions (25). Teams working to improve care typically do not have the necessary skills to set up a robust measurement approach, and often end up collecting or extracting data that is inadequate to address the above needs. It is necessary to understand how to develop measurement training, support and infrastructure required to operate such approaches in routine healthcare practice (27). Further work is also needed to advance methodology capable of evaluating complex interventions in complex systems that enable timely and reliable assessment of system performance. It is also necessary to learn how best to report and synthesise evaluations to support future improvement efforts (28, 29), and to establish the best approaches to share learning and experiences between settings (30).

The areas in this cluster are closely related—improvement initiatives and research studies need to be conducted within existing healthcare systems to understand how improvements will be integrated into routine care, and how they compete against other priorities and workstreams. Leaders who select improvement goals and approaches need to work with what is already present in the system (skills, capacity, resources, experience, relationships, tensions). They need to understand the culture and context for improvement, including past experiences and their results, within each setting. While these areas are closely related, tensions can also exist between them. For example, evaluation and measurement agendas can conflict with improvement when the launch of an intervention is prioritised over establishing a clear baseline.

## Cluster 2—aligning improvement efforts

### The interdependence of system levels

As well as considering the requirements of any individual improvement effort it is necessary to consider improvement efforts within the wider health system. Any specific initiatives are part of a wider system that is influenced at individual, micro- (team or department), meso- (organisation) and macro-system (policy and regulation) levels (31). Initiatives targeting one level of the system will interact with other initiatives and other system levels. While some may be aligned, the misalignment of others causes tension and duplication which is often experienced most acutely by those at the sharp end of care delivery (11). Work is required in practice and research to understand the complex array of initiatives taking place and to inform the orchestration of such alignment, balancing the need for entrepreneurship and coordinated direction.

### The interactional work of improvement

Fundamental to all improvement efforts is the human dimension of change including issues of relationships, trust, and motivation to change. These issues are critical for successful improvement but are often under recognised in teaching, planning and evaluation of improvement approaches. The interpersonal and interactive aspects of improvement often require skills such as negotiation, facilitation, building relationships and the ability to create a trusting environment (32). These are essential for sharing knowledge, seeking agreement, changing behaviours, and challenging the status quo resulting from organisational hierarchies and vested interests (33). It is necessary to understand the time, skill and support required for the interactional work of improvement, and the implications this has for the design, conduct and evaluation of improvement initiatives, improvement approaches, and research studies.

### Enhancing the value of improvement practice and research

An important consideration for the improvement community is where to invest resources. Not all improvement or research activities are of equal value and some may be wasteful or harmful (34). Given the finite resources in terms of time, capacity and expertise, these need to be used as effectively as possible. Effective use of resources needs to be considered within individual improvement initiatives and research studies, within organisations, and across the whole system that influences healthcare improvement (35). Study designs that can address system complexity and learn from multiple variables in single studies need to be more extensively deployed (36). There is a need to understand what resources are already in place, how the value of investments can be assessed, and how to disincentive low value or duplicative work whilst incentivising high value, synergistic work.

The three areas in this cluster have the potential to facilitate or hinder any improvement approach depending on whether they are aligned with, or disruptive to, “normal” work.

## Cluster 3—advancing the contribution of the improvement community

### Seeking synergies between knowledge, research, and practice to achieve improvement

The two-way relationship between research and practice is critical in advancing contributions from the improvement community. However, currently practitioners and researchers tend to work in relative isolation from each other and there is a recognised gap between the production of evidence and new knowledge and its use in practice (37). Practitioners rarely have the time or inclination to keep up to date with the latest research findings, and researchers typically design their studies to address issues of academic interest rather than practical importance. There is a need to understand how best to achieve synergy between research and practice and to learn what active approaches are needed to support the mobilisation of knowledge into changes in practice (38). Improved infrastructure, development of new roles and career pathways, and incentives to enhance relationships and partnerships could benefit everyone: practitioners could benefit from access to knowledge to inform improvement attempts, and researchers need access to practice settings where improvement efforts take place to generate useful new knowledge (39). Such access enables two-way exchange of needs, ideas and theories to inform future improvement efforts, and to generate refined theories, actionable findings, and ultimately to improve the quality of patient care.

### Ways of knowing and doing

Advancing the contribution of the improvement community depends on how well people are able to work together when they come from diverse backgrounds, disciplines and specialities. This depends on understanding how collaboration can take place between people who hold different world views and perspectives, who work to different standards, and prioritise different outputs and outcomes. Differences between stakeholders in ways of knowing and doing can make such synergies challenging to achieve (40). Particular fields have well founded traditions of enquiry that have enabled knowledge generation within them—as exemplified by debate regarding the hierarchy of evidence and the role of randomised controlled trials in improvement (7, 41)—but these traditions may hinder exchange of knowledge between fields. For example, quality improvement approaches tend to emphasise the importance of building local capacity and drawing on internal knowledge and expertise to guide improvement efforts whilst implementation science approaches tend to rely on external expertise to design and then disseminate interventions. These differences in perspective reflect distinctions in the origins and philosophy of each approach regarding what types of knowledge are valued and the extent to which people working in the system are viewed as having agency or requiring assistance. Whilst both quality improvement and implementation science have value to offer the improvement community, these fundamental differences can create tension and conflict between different perspectives. It is important that such philosophical distinctions are understood and valued to facilitate meaningful dialogue and exchange between different disciplines and perspectives. It takes time to build a depth of understanding and appreciation of how different disciplines and approaches can work together for the benefit of the improvement community, and this can be supported by reflective practice and empirical enquiries to explore the benefits and limitations of different approaches.

### Building the community

Building a community that is skilled in improvement practice and research, and able to converse across diverse professional, patient and academic perspectives, is critical. The complexity of healthcare improvement makes contributions from a diverse range of disciplines valuable, but also challenging to teach, study and learn (42). Many questions remain around how to build such capacity and capability. For example, it is not yet clear what breadth and depth of improvement knowledge and competencies are optimal across a healthcare system, or how best to develop and distribute expertise across the workforce. There is also a need to understand how to build career pathways for the improvement community, and how these fit with existing pathways in healthcare organisations, academia, and policy. If the improvement community is going to extend its contribution outside of dedicated specialists and aficionados it must be able to communicate the value of the field to external stakeholders including politicians, funders, healthcare leaders. Tackling this issue is important for the future of improvement and, therefore, should influence the design of communication, education and professional development for academics, practitioners, local and system leaders, policy makers, patients and members of the public (43).

## Distinguishing features of improvement science

We propose there are four features that collectively define and distinguish the field of improvement science: the conceptualisation of health services as complex social systems; the promotion of a holistic person and system perspective; a focus on the practical applicability of knowledge; and the acknowledgement of both the necessity and challenges of engaging people from diverse backgrounds to collaborate.

These distinguishing features, whilst they might individually share overlaps with other fields of study, collectively start to illustrate the scope of interest and nature of enquiry of improvement science field in transcending traditional boundaries of academic and health service practices.

First, as illustrated by the ten areas of enquiry, improvement science *conceptualises health services as complex social systems*, defined by the agency of people within the system, the interconnectedness of system parts, and the dynamics of people and systems that change over time and are influenced by historic events (44).

Second, and as a consequence of adopting a complex systems view, improvement science encourages a move away from the relative comfort of focusing on single issues, interventions or aspects of quality at a time, or working in controlled or remote environments—as often practiced by academics, innovators, policy makers and service planners—towards a holistic view of how different aspects of the system work together in routine practice. In doing so, improvement science *promotes a holistic person and system perspective*, viewing the system as it is experienced by those embedded in the system, namely the staff who deliver and the people who receive health services (45). By placing primacy on the perspective of those embedded in the systems it is necessary to recognise that work is often conducted in imperfect and high-pressured environments, with competing demands and priorities that cannot easily be resolved. Central to adopting a holistic system perspective is the recognition of the causes and consequences of variation, and how these can be understood and responded to (26).

Third, *the practical relevance and application of knowledge is a central concern.* Knowledge about how to improve any given system can only emerge from intervening in the system and learning from and responding to what emerges (46). Improvement science must move beyond descriptions of enablers and barriers, and demonstrations of one-off successes or isolated pockets of excellence, to clearly show how such knowledge can be applied to achieve improvements reliably and equitably across diverse contexts and settings. In doing so improvement places greater emphasis on the practical and context specific issues of translating, mobilising and applying knowledge, than in any inherent value of knowledge in its own right. Advances in how we can improve the way we improve require a critical exploration of the relationships between knowledge and practice, between academic and healthcare organisations, and between science and society.

Finally, as the breadth of the improvement landscape indicates, an enormous range of knowledge and expertise is required to advance the understanding and practice of improvement. Meaningful progress—where we are learning from each other and building on prior evidence and experience—depends heavily upon collaboration and co-production. However, it is important to acknowledge both *the necessity and challenges of engaging people from diverse backgrounds to work together collaboratively*. Whilst each area of enquiry in the improvement landscape is of importance, we believe the need to collaborate to collectively address the breadth of issues in a synergistic manner is the greatest challenge of all. Knowing that different areas of expertise exist is a start, but truly valuing and understanding diverse and often conflicting perspectives, and the ability to communicate across (professional and disciplinary) language and cultural barriers to build trusting and respectful relationships is required if we are to work synergistically across the improvement landscape.

## Where next for practice and research?

To our knowledge this is the first attempt to outline the breadth of areas of enquiry that are of importance to the improvement community and the field of improvement science. We believe this outline serves as a foundation stone for defining the breadth and interests of the field, and for further exploring how it overlaps with and is distinguished from other fields of study and practice.

Bringing the breadth of areas of enquiry together into a single outline highlights the diverse experience and expertise required to *achieve improvements in practice*, *align improvement efforts*, and *advance the contribution of the improvement community*. Whilst each of the areas, clusters and questions presented maybe very familiar to some people, there are very few people who are expert or even conversant in all of the areas presented. In this respect, we hope this paper can act as a call to the improvement community to look beyond their traditional areas of interest and to start to explore how insights and expertise from different areas can support and enhance each other, and in turn advance the study and practice of healthcare improvement. The potential applications of the outline of the improvement landscapes for different user groups is considered in Table 2.

**Table 2:** Potential application of the outline of the improvement landscape for various user groups.

User group	Potential application of the outline of the improvement landscape
Individual teams/practitioners	• Outline the breadth of issues that influence every improvement initiative. • Provides a structure for improvement teams to make explicit considerations regarding the design, execution and evaluation or improvement initiatives, and to highlight influences that are out of their control. • Identifying such issues at the outset of an improvement initiative can increase chances of success, or if used to review initiatives retrospectively may reveal areas of important learning.
Researchers	• Identify where studies and programmes of research sit within the wider improvement landscape. • Identify how studies contribute to advancements of knowledge within specific frontiers. • Provide greater awareness of where else work is taking place, which may in turn lead to new collaborations, reframing of research questions, or changes to study design.
Educators	• Guide the development of teaching materials and curriculum for practitioners and researchers, providing awareness of the multiplicity of factors that influence and are of interest in healthcare improvement. • Enable students to situate their learning within the wider improvement landscape • Prepare students to better anticipate and understand problems and challenges that they encounter, and know where to turn to further their learning.
Policy makers and organisational leaders:	• To appraise the breadth of factors that need to be considered in designing improvement policy interventions and strategies • To inform the effective deployment resources • To design policies and healthcare systems that support improvement as a core activity
Research and funding bodies:	• Assist funders in reviewing applications and allocating funding • Consider the balance or investment across the breadth of the improvement landscape. • Identify which areas under invested in and hampering the advancement towards community goals. • Help reprioritise and incentivise research in under resourced areas.

We invite others to refine and advance mapping of the improvement landscape by identifying additional gaps and increasing contributions from diverse perspectives. We recognise the limitation that the authors provide a European and North American perspective and, whilst international conference communities were engaged in its development, the work would be enhanced by further exploration from wider geographical and sociodemographic perspectives.

We hope the outline of the improvement landscape starts to provide a common language for the diverse improvement community, enabling people to transcend disciplinary interests and constraints, and to consider how, collectively, we can improve health and care.

## Data Availability

The original contributions presented in the study are included in the article/Supplementary Material, further inquiries can be directed to the corresponding author.
