# Supplementary material for: What is improvement science, and what makes it different? An outline of the field and its frontiers
Source: Front Health Serv. 2025 Feb 20;4:1454658. doi: 10.3389/frhs.2024.1454658 (PMC11884242; doi:10.3389/frhs.2024.1454658)
Supplement: Supplementary file 1 [file Datasheet1.pdf]

Supplementary Material – Process of developing the conceptual map and identifying the frontiers of improvement.

Origins and inspiration:

The Conceptual Map and Frontiers of Improvement as presented in this paper have been developed throughout an iterative process, which has been formally started in 2016 by a multidisciplinary group of Improvement Science Fellows from UK and Sweden, together with other improvement scientists from Europe and North America (see figure 1), with the objective to coordinate efforts to advance the field of healthcare improvement.

This work was inspired by initiatives in other fields to outline the grand challenges and frontiers of knowledge to inspire and guide their research communities. This includes mathematical problems identified by David Hilbert in the 1900s which set the course for much of the mathematical research of the 20th century,(1) and which has since been updated with the Millennium Problems.(2) Similarly, the 2003 position piece "Beyond the Molecular Frontier" outlines the progress made, and the grand challenges still remaining, in the chemical sciences.(3) This work was a particular source of inspiration for how it called for different disciplines within academic chemistry and chemical engineering to pull together to solve problems of societal importance. These examples inspired us to set out on a similar journey to identify the frontiers of improvement in health care.

The breadth of academic and practitioner backgrounds engaged with the improvement community is exemplified by the authors of this paper. Supported by the Health Foundation (UK) (4) and Vinnvård (Sweden),(5) the authors have participated in an international Improvement Science Development Group which brings together experts from a breadth of academic disciplines and practitioner backgrounds.(6) A common characteristic of people in this community is that they inhabit hybrid roles, bringing both in-depth knowledge from specific academic disciplines and first-hand experience of working with frontline practitioners and patients. This community provided us (the authors) with opportunities for detailed discussion and exploration over an extended period of time (2011 - 2018). All members of the improvement science fellowship groups in the UK and Sweden were invited to participate in the core group of authors leading this work. Those involved in this paper represent all cohorts from both countries. All other fellows were involved in group discussions and workshops.

The authors have experienced the challenges of transferring knowledge and understanding held by different fields and disciplines, with expert academic knowledge frequently perceived as inaccessible, fragmented, and difficult to understand by those from other sectors. At the same time, we developed a deeper respect for each other's disciplines and perspectives, recognizing that all who seek to improve healthcare can benefit from this diversity of backgrounds and academic disciplines.

Data collection and analysis

The development of the conceptual map and frontiers was led and coordinated by the authorship group, with consultation and discussion with members of the wider improvement community. The overall process of development is presented in figure 1.

Within authorship meetings, there was a deliberate alternation between individual contributions, small groups and whole group discussions, in order to ensure that all voices were heard and to give space for conflicting opinions to be explored. Group techniques, such as brainstorming to generate ideas, nominal group technique and voting were used to gather and appraise ideas and to reach consensus. Data were gathered through notes, flipcharts, sticky notes and by collecting any document produced or edited by the participants during the meetings.

Feedback was obtained from the wider improvement community during national and international workshops, events and conferences involving audience with different roles within the health system (e.g. academics, healthcare professionals, quality improvement experts, managers, policy makers, patients etc.).

During conferences and other events, authors provided the audience with an overview of the current version of the Frontiers of Improvement and collected feedback through the on-line platform Mentimeter,(7) which allowed electronic submission of answers up to 140 characters in length, with individual participants able to enter as many responses as they wished. During live events notes were also taken by authors about questions and comments from the audience.

Throughout this process emerging concepts were progressively refined, tested and consolidated. In doing so authors made a concerted effort to value the different perspectives and vocabularies of each team member, investing time to understand the diverse theories and disciplines they each work with, rather than negotiating away nuance in favour of quick compromise. They paid particular attention to the use of language that was accessible and enabled diverse disciplines to engage.

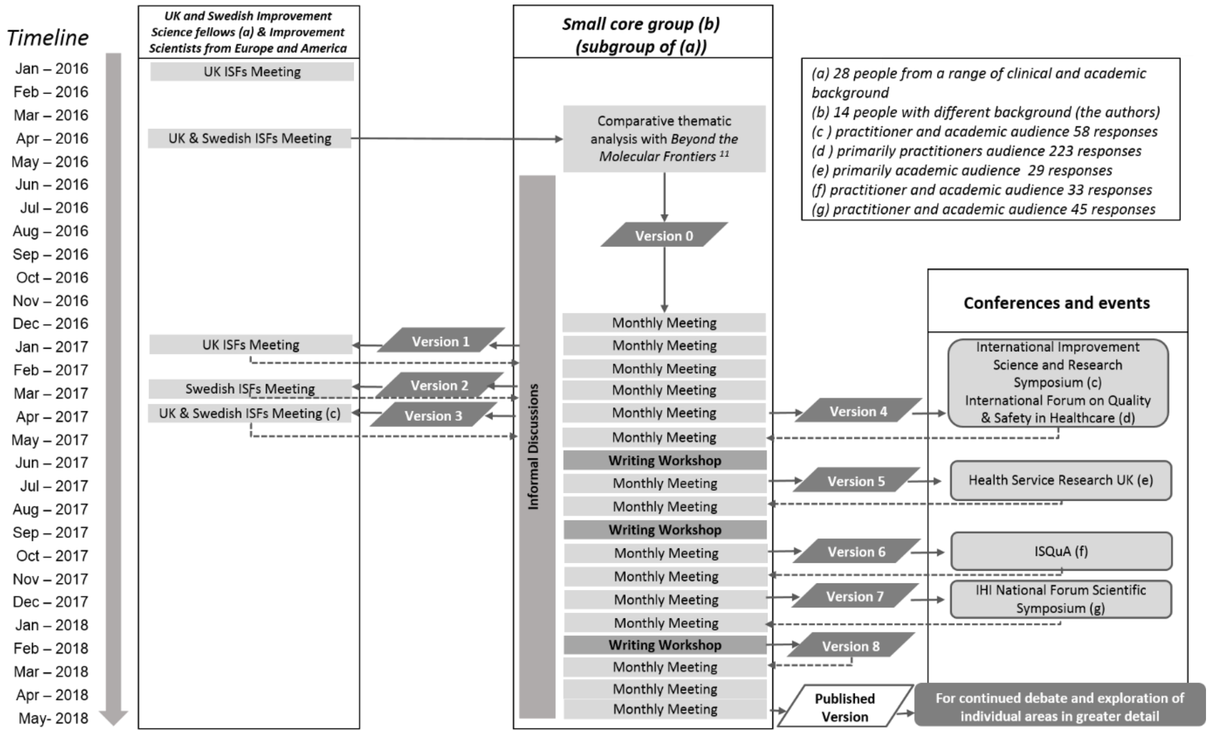

FIGURE 1 THE PROCESS BY WHICH THE CONCEPTUAL MAP AND FRONTIERS OF IMPROVEMENT, PRESENTED IN THIS PAPER, WERE DEVELOPED.

Development timeline

In January 2016 during the UK Improvement Science Frontiers Meeting two authors (JR and TW) presented the idea of articulating the frontiers of improvement, drawing on inspiration from the fields of chemistry and mathematics. The workshop session was used to conduct an initial brain storm of issues at the frontiers of improvement in healthcare.

The theme emerging from the January 2016 meeting were then presented to the International Improvement Science Development Group Meeting (April 2016) involving UK and Swedish Improvement Science Fellows (28 people from a range of clinical and academic background). Feedback was collected from participants in response to the themes presented, and to the invitiation to present new as yet identified themes, areas and questions. These data were then analysed using comparative thematic analysis. Resulting themes where comparatively analysised by JR with the range of themes identified in Beyond the Molecular Frontiers to reflect on the breadth of issues covered and identify any missing areas from a strategic scientific perspective.(3)

This process led to the first conceptualization of the main areas and major challenges that needed to be addressed in order to improve the way in which improvement is delivered, evaluated and studied (Version 0). This version was discussed by the authors during the first online monthly meeting (December 2016) and then refined using online tools to share and edit documents. A new version (Version 1) was presented at the UK Improvement Science Fellows Meeting held in London in January 2017 (28 people). During this workshop feedback was gathered on the proposed areas and challenges, with specific request for identification of missing areas or challenges, or language or concepts requiring clarification or amendment. Space was provided in the face to face meeting for debates about such issues to occur. As well as written feedback (post-it notes, flip charts) notes were taken by members of the authorship group. Emerging themes were analysed and discussed during the following online meeting as to how incorporate them. The resulting refined version (Version 2) was presented and discussed by the Swedish Improvement Science Fellows Meeting held in Stockholm in March 2017. Following the same process, data were analysed, and changes discussed by authors. A new version (Version 3) was presented in April 2017 at joint meeting between the UK and Swedish Improvement Science Fellows. This resulted in further minor modifications to descriptions of the areas and frontiers, but overall there was a good level of agreement amongst the groups with no additional themes, areas or challenges identified, suggesting consensus was being reached amongst the UK and Swedish fellowship group (Version 4).

The authorship group then sought input from the wider improvement community at the International Improvement Science and Research Symposium and at the International Forum on Quality and Safety in Healthcare. During these events, authors gave a presentation about the Frontiers identified so far and the audience were invited to answer three questions: i) “What would you most like the field of improvement science to achieve in the next 20 years?” ii) “What are the main things that need to progress to achieve that?” And iii) “What challenges do you see to achieving this progress, and how might they be overcome?”. Responses from the audience were shared through the on-line platform Mentimeter.(7) This method ensured that the debate was kept open with the encouragement to audience members to identify new ideas and gaps in the presentation, as well as the opportunity to agree with what had already been proposed.

The International Improvement Science and Research Symposium involved primarily a practitioner and academic audience, collating 58 responses. At the International Forum on Quality and Safety in Healthcare 223 responses were collected. Comparative thematic analysis of these data was conducted, looking for areas of agreement and endorsement of the existing frontiers, and for new areas and challenges to widen our view of the landscape.

The analysed data was initially discussed amongst all authors in the next online monthly meeting (May 2017) and then in greater depth at a 3 day face-to-face workshop (19th-21st June 2017 Stockholm). Eight authors joined the meeting in person (JR, GA, NA, YJ, JT, SC, TW, KP), while others participated via Skype. JR and GA presented a summary of the latest version of the Frontiers conceptualization, alongside the themes emerging from the analysis of data collected from the two international events. This presentation was followed by a group discussion about the alignment and gap amongst old and new themes and proposals of frontiers reconceptualization. The main Improvement Frontiers identified so far were then reported on a flipchart and participants were invited to vote how confident/comfortable they were with the current Frontiers conceptualization and to add any comment they had using sticky notes. Participants then split in pairs to address comments emerging from previous discussions and the voting exercise.

At the end of this workshop a first full draft of the Conceptual Map and Frontiers (Version 5) was produced, which was presented at the Health Service Research UK conference (July 2017) involving mainly an academic audience. During this event 29 responses were collected and analysed following the same process as for the previous conferences.

Emerging data were discussed in the following online monthly meeting (August 2017) and in the second workshop, which was held in London in September (4th-6th) 2017. This workshop was particularly focused on working on the paper draft to refine messaging and incorporate further feedback. Ten authors joined the meeting face-to-face (JR, GA, NA, DH, YJ, JT, SW, TW, MR, KP), while the rest participated using Skype. S After these 3 days, a revised version (Version 6) of the conceptual map and frontiers was produced.

Version 6 was presented in October 2017 at the ISQua conference in Kuala Lumpur, Malaysia. During this international conference, the Conceptual Map and Frontiers were presented, and data collected and analysed using the same process as for the other events. Due to the wider event's theme there was a focused interest amongst participants on the spread and sustainability of improvement, but analysis of areas of importance to this group revealed alignment with areas across the entire conceptual map. Emerging themes were discussed in the next online monthly Meeting and a revised version (Version 7).

The final opportunity for input and data gathering was through the IHI National Forums Scientific Symposium (December 2017, Orlando, USA). The feedback gathered in this event was analysed and discussed during the last authors workshop held in February (21st -23rd) 2018 in London. This meeting aimed at addressing further comments raised by contributors and authors over the previous 6 months and further refining the wording, and presentation of the conceptual map and frontiers. Twelve authors joined in person (JR, GA, NA, KP, SW, TW, MR, YJ, CS, SC, DH) while others joined via Skype.

At the end of the meeting, the revised version of the Conceptual Map and Frontiers (Version 8)

References

1. Hilbert D. Mathematical problems. Bull Amer Math Soc. 1902;8(10):437–79.

2. Devlin K. The Millennium Problems: The Seven Greatest Unsolved Mathematical Puzzles Of Our Time. Illustrated edition. New York: Basic Books; 2003.

3. Beyond the molecular frontier: Challenges for chemistry and chemical engineering. National Research Academies Press, Washington 2003.

4. Improvement Science Fellowships - The Health Foundation [Internet]. [cited 2022 Feb 5]. Available from: https://www.health.org.uk/funding-and-partnerships/fellowships/improvement-science-fellowships

5. Vinnvård Improvement Science Fellows - Vinnvård [Internet]. [cited 2022 Feb 5]. Available from: http://www.vinnvard.se/en/vinnv/

6. Improvement Science Development Group. Secondary Improvement Science Development Group. [Internet]. [cited 2020 Sep 22]. Available from: https://www.health.org.uk/newsletter/improvement-science-finding-evidence-support-healthcare-improvement.

7. Interactive presentation software [Internet]. Mentimeter. [cited 2022 Feb 3]. Available from: https://www.mentimeter.com/
